# Hospital Vacancy, Etc.

**Published:** 1876-01

**Authors:** 


### Hospital Vacancy.

We are requested to state that the position of the Resident Physician to the Buffalo General Hospital will be vacant on the first of March, 1876. The position will be filled by means of a competitive examination, and all applicants who intend entering the examination should hand their names and credentials to Dr. Conrad Diehl, Secretary of the Hospital Staff, on or before February 22d, who will notify them of the time and place of examination. By a recent regulation the term of Resident Physician is limited to one year.

The *Leavenworth Medical Herald* and the *Kansas City Medical Journal* have suspended.——The *American Psychological Journal* is now edited by Dr. Allen McLane Hamilton, assisted by an able corps of associate editors. The department relating to Medico-Legal subjects has been transferred to the *Sanitarian.* The *Chicago Journal of Nervous and Mental Diseases* will hereafter drop the word *Chicago* from its title, and will be published simultaneously in Chicago and New York. Messrs. G. P. Putman's Sons have assumed its publication in New York. The Editorial management remains unchanged, but the co-operation of Dr. Wm. A. Hammond, of New York, Dr. S. Weir Mitchell, of Philadelphia, and Dr. E. H. Clark, of Boston, has been secured in conducting the Journal.——Owing to a press of other matter the department of *Medical Notes* has been crowded out for the past few months.

